# SARS-CoV-2 and Emerging Variants: Unmasking Structure, Function, Infection, and Immune Escape Mechanisms

**DOI:** 10.3389/fcimb.2022.869832

**Published:** 2022-05-12

**Authors:** Jiaqi Li, Huimin Jia, Miaomiao Tian, Nijin Wu, Xia Yang, Jianni Qi, Wanhua Ren, Feifei Li, Hongjun Bian

**Affiliations:** Shandong Provincial Hospital Affiliated to Shandong First Medical University, Jinan, China

**Keywords:** COVID-19, SARS-CoV-2, spike protein, receptor, variants

## Abstract

As of April 1, 2022, over 468 million COVID-19 cases and over 6 million deaths have been confirmed globally. Unlike the common coronavirus, SARS-CoV-2 has highly contagious and attracted a high level of concern worldwide. Through the analysis of SARS-CoV-2 structural, non-structural, and accessory proteins, we can gain a deeper understanding of structure-function relationships, viral infection mechanisms, and viable strategies for antiviral therapy. Angiotensin-converting enzyme 2 (ACE2) is the first widely acknowledged SARS-CoV-2 receptor, but researches have shown that there are additional co-receptors that can facilitate the entry of SARS-CoV-2 to infect humans. We have performed an in-depth review of published papers, searching for co-receptors or other auxiliary membrane proteins that enhance viral infection, and analyzing pertinent pathogenic mechanisms. The genome, and especially the spike gene, undergoes mutations at an abnormally high frequency during virus replication and/or when it is transmitted from one individual to another. We summarized the main mutant strains currently circulating global, and elaborated the structural feature for increased infectivity and immune evasion of variants. Meanwhile, the principal purpose of the review is to update information on the COVID-19 outbreak. Many countries have novel findings on the early stage of the epidemic, and accruing evidence has rewritten the timeline of the outbreak, triggering new thinking about the origin and spread of COVID-19. It is anticipated that this can provide further insights for future research and global epidemic prevention and control.

## Introduction

Coronaviruses are genotypically divided into four genera (alpha, beta, gamma, and delta) (Woo et al., 2010), of which α- and β-coronavirus can be major human pathogens by crossing the animal-human barrier (Cui et al., 2019). Up to the present, there are seven known species of human coronaviruses: 229E (α-CoV), NL63 (α-CoV), OC43 (β-CoV), HKU1 (β-CoV), MERS-CoV (β-CoV), SARS-CoV (β-CoV), and SARS-CoV-2 (β-CoV) (Qiang et al., 2020). Specifically, SARS-CoV-2 is a single-stranded, positive-sense RNA virus, belonging to the Sarbecovirus subgenus (Zhou et al., 2020). Like SARS-CoV and MERS-CoV, SARS-CoV-2 is classified as a zoonotic β-coronavirus that possibly originated from bats and was transmitted to humans through distinct intermediate hosts (Hu et al., 2021). As regards the origin of SARS-CoV-2, there is almost no theory. However, the most probable one is that it has a natural, zoonotic origin. Zoonotic viruses, including influenza viruses and coronaviruses, are most likely derived from wild animals and remain a continuous threat to humans. To date, no studies have completely elucidated any potential natural hosts or intermediate hosts for SARS-CoV-2. Based on phylogenetic analysis, SARS‐CoV‐2 is closely correlated with SARS‐CoV and far from MERS‐CoV and its nucleotide homology with SARS‐CoV and MERS‐CoV is 79 and 50%, respectively (Hu et al., 2021). Thus, comparative analysis of SARS-CoV-2 and SARS-CoV will contribute to further clarifying the pathogenesis of SARS-CoV-2.

The SARS-CoV-2 genome is between 26 kb and 32 kb in size and 60-140 nm in diameter (Khan et al., 2020). SARS-CoV-2 harbors 15 open reading frames (ORFs) encoding nonstructural proteins (NSP1-16), structural proteins (N, M, E, S proteins), and accessory proteins (ORF3a, 3b, 6, 7a, 7b, 8, 9a, 9b and 10) (Liu et al., 2014; Zinzula, 2021). The replicase genes located in the first two-thirds of the genome are first translated into two large polyproteins pp1a and pp1ab, which are then processed into 16 NSP *via* proteolytic cleavage by the viral main protease (M^pro^, NSP5, or 3CL^pro^) and a papain-like protease (NSP3, PLpro). The remaining one-third of the genome contains ORFs for the structural proteins, namely the spike (S), envelope (E), membrane (M), and nucleocapsid (N) proteins (Liu et al., 2014; Majumdar and Niyogi, 2021).

During cellular infection by SARS-CoV-2, the trimeric spike (S) proteins located on the viral surface enter the host cell through the ACE2 receptor and TMPRSS2 (Lu et al., 2020) (Hoffmann et al., 2020; Hatmal et al., 2020). ACE2 is a type I membrane-bound protein that is widely expressed in the heart, lung (especially in type 2 pneumocytes), kidney, digestive organs, liver, testis, brain, and vascular endothelium (Hamming et al., 2004). This discrepancy remains hard to interpret the multi-organ tropism of SARS-CoV-2. Remarkably, both SARS-CoV-2 and SARS-CoV use ACE2 as a receptor in host cells (Hoffmann et al., 2020). However, they showed significant differences in epidemiological, target organs, pathogenetic and clinical characteristics. Previous work has indicated that SARS-CoV-2 may depend on co-receptor or other auxiliary membrane proteins to invade the human host and cause severe disease.

Mutations are integral parts of the virus life cycle and rarely significantly affect outbreaks. Nevertheless, as an RNA virus, SARS-CoV-2 has a high mutation rate and recombination events due to the low fidelity of RNA polymerase. The genetic evolution of SARS-CoV-2 occurred in a continuous adaptation to new human hosts, resulting in mutant variants that forced many countries have to endure the second or third wave of outbreaks. These variants not only seem to spread more effectively in susceptible hosts than the virus from the initial outbreak but also may be more resistant to naturally acquired or vaccine-induced immunity (Cai et al., 2021).

Overall, we summarized the latest research, demonstrated the epidemiological characteristics, basic virology including genome structural characteristics and potential receptors, and analyzed the effects of different variants on transmission and virulence. Subsequently, we elaborated on the clinical manifestations of COVID-19, including attacks and pathogenic mechanisms on various organs of the human body, along with classic and new therapeutic approaches.

## Epidemiology of All Period

### The Pandemic’s Status

Three highly pathogenic coronaviruses, SARS-CoV, MERS-CoV, and SARS-CoV-2 frequently cause severe respiratory distress and multiple organ failure with high mortality. The third highly pathogenic strain of coronavirus, SARS-CoV-2, was reported for the first time in late December 2019 in the Hubei province in China and rapidly spread and broke out in the world (Zhu et al., 2020). The spread of SARS-CoV-2 is classified by the World Health Organization (WHO) as a public health emergency of international concern, and pneumonia caused by SARS-CoV-2 has been designated as coronavirus disease 2019 (COVID-19) ([[NoAuthor]]; Team EE, 2020). SARS-CoV-2 is highly contagious due to its direct and rapid human-to-human transmission, especially in comparison to the SARS-CoV and MERS-CoV coronaviruses, which were declared a pandemic by the World Health Organization on March 11, 2020 (Jee, 2020).

Countries that were hit hard by this outbreak in terms of the total number of individuals infected include (in descending order): the United States, India, Brazil, France, Germany and the United Kingdom (WHO, 2022a). The current ongoing pandemic wave has quickly spread to other parts of Asia and subsequently to Europe and other countries, and has caused countless morbidity and mortality worldwide. Among these, America had the largest number of SARS-CoV-2 confirmed cases and deaths and was recognized as the epicenter of the pandemic. This unexpected infection is threatening human health and, consequently, devastating the global economy.

### Early Stage of Transmission in Different Regions

Wuhan, the hardest-hit area of China, the first recorded cases were reported in December 2019 and then reached an epidemic peak in February 2020. The emergency response and massive intervention measures were implemented by the Chinese governments at all levels to block the epidemic spread. Following a strict lockdown policy, the epidemic situation was generally under control in China, while the outbreak outside Mainland China is reported to begin on a large scale.

The first documented case in America was in Snohomish County, Washington, on January 20, 2020 (Holshue et al., 2020). The patient was a person who returned from a trip to Wuhan on January 15, 2020. Subsequently, the outbreak spread throughout all 50 states in America in early March, of which New York remains the region most severely affected to date. The genomic epidemiological of SARS-CoV-2 demonstrates that the initial COVID-19 outbreak on the west coast of the United States was mainly derived from Chinese isolates, while the pandemic in New York and the east coast of the US came from European isolates (Zhang et al., 2020).

In Europe, the first confirmed case had contact with parents from Wuhan (Böhmer et al., 2020). The patient was reported to be infected with SARS-CoV-2 on Jan 26 which resulted in 16 subsequent cases of infection (Böhmer et al., 2020). Despite the implementation of multiple control measures, the epidemic continued to spread across Europe and North America and reached Africa (Egypt) on February 13, 2020. As for sub-Saharan Africa, the first confirmed case appeared in Nigeria (Giandhari et al., 2020). As the last continent where the pandemic spread, South America confirmed its first case on February 25, 2020. By then, no continent in the world was spared (Elizondo et al., 2021).

### Novel Evidence of Epidemiological Origin

As the research continues to deepen, although the exact origin remains debatable, there is some small but increasing evidence regarding the source of this outbreak. Recently, many countries have made some discoveries about the early COVID-19 epidemic, and the continuous emergence of earlier cases has triggered deeper reflections on the origin and spread of the virus, which may rewrite the global timeline of the COVID-19 epidemic.

Outside of China, Italy was the first European country to be very strongly impacted by COVID-19 with the first autochthonous case identified in Lombardy on February, 21st, 2020 (Onder et al., 2020). Based on the complete genomic characteristics and phylogenetic analysis, SARS-CoV-2 emerged in Northern Italy a few weeks before the first case was reported. In a previous study, as early as mid-December 2019, environmental surveillance has unambiguously demonstrated that SARS-CoV-2 is present in untreated wastewater of the Milan area, and its concentration is the same as that of samples collected in the later stages of the pandemic (La Rosa et al., 2021). In addition, an oropharyngeal swab sample collected on December 5, 2019, suggested that a 4-year-old boy with no travel history in the Milan area tested positive for SARS-CoV-2, which was 3 months earlier than Italy’s first reported case (Amendola et al., 2021). After that, an international research team led by the University of Milan in Italy reported that they found the new coronavirus gene sequence in a biopsy sample from a young female dermatologic patient on November 10, 2019 (Gianotti et al., 2021). This result advanced the appearance of the Italian “Patient Zero” from January 30, 2020 to November 2019. This finding is of considerable epidemiological significance as it substantially enhances our understanding of time and the map of the SARS-CoV-2 transmission routes.

Accumulating evidence has proved that in December 2019 the virus has spread throughout Europe and was misdiagnosed as cases of influenza (Deslandes et al., 2020). According to research, in many areas where wastewater has been sampled, including France (Paris), Spain (Murcia), and three cities and regions in northern Italy (Milan/Lombardy, Turin/Piedmont, and Bologna/Emilia Romagna), SARS-CoV-2 RNA has been detected in the effluent before the local government announced the first case of COVID-19, reflecting that the possible presence of large numbers of asymptomatic carriers and symptomatic patients who were shedding viral RNA prior to the first reported case (Randazzo et al., 2020; La Rosa et al., 2021). A French study has proved the fact through retrospective analysis, and a French with no aetiological diagnosis for hemoptysis tested positive for SARS-CoV-2 (Deslandes et al., 2020). The patient was hospitalized on 27 December 2019 and presented clinical features and radiological patterns frequently observed in Chinese and Italian cohorts previously, indicating that the virus was already spreading throughout the French population in late December 2019 owning to the lack of recent foreign travel (Deslandes et al., 2020).

In accordance with the recent SARS-CoV-2 genome diversity analysis results, all sequences as of the end of 2019 shared a common ancestor and the virus has existed in the human host for a considerable time before it was identified (van Dorp et al., 2020).

### Transmission Routes of SARS-CoV-2

As we all know, respiratory droplets and direct contact are the main pathways of SARS-CoV-2 transmission. In common with other CoVs and influenza, SARS-CoV-2 can cause infection by invading mucosa of the eyes, nose, or mouth when infected persons cough or sneeze. Previous work has demonstrated that aerosol and fecal-oral routes also transmit SARS-CoV-2 (Wang et al., 2020). Based on evidence that the SARS-CoV-2 virus could remain viable in an aerosol for at least 3 hours in a closed environment (van Doremalen et al., 2020).

Although the precise mechanism of SARS-CoV-2 transmission is still unclear, with the deepening of research, other transmission routes have been hypothesized. There is growing evidence that, though extremely rare, vertical transmission *in utero* is possible and its underlying mechanism is possibly correlated to the transmission of the well-known SARS-CoV-2-related inflammatory status to the fetus (Fenizia et al., 2020). A recently published study has also shown that SARS-CoV-2 may not be transmitted during childbirth, as it was not detected in vaginal secretion, breastmilk, neonatal throat swab, umbilical cord blood, and amniotic fluid of pregnant women (Mohseni et al., 2020). However, due to the up-regulation of the ACE2 receptor expression, the fetal liver can be a target organ for SARS-CoV-2 infection during pregnancy (Mohseni et al., 2020). Therefore, the effect on the placenta and the intrauterine vertical transmission potential of SARS-CoV-2 need to be further carefully explored, and its adverse consequence for maternal and infant should not be underestimated. In addition, body fluids are risk factors for viruses to invade the body (Mohseni et al., 2020).

## The Genome Structure General Characteristics of SARS-CoV-2

The full-length genome of SARS-CoV-2 is 30 kb, including the 5′-region encoding for non-structural proteins and the 3′-region encoding for structural proteins. It has 14 open reading frames (ORFs) coding for 27 proteins: structural proteins, nonstructural proteins, and accessory proteins (Hatmal et al., 2020).

### Non-Structural Proteins

**Figure 1:**
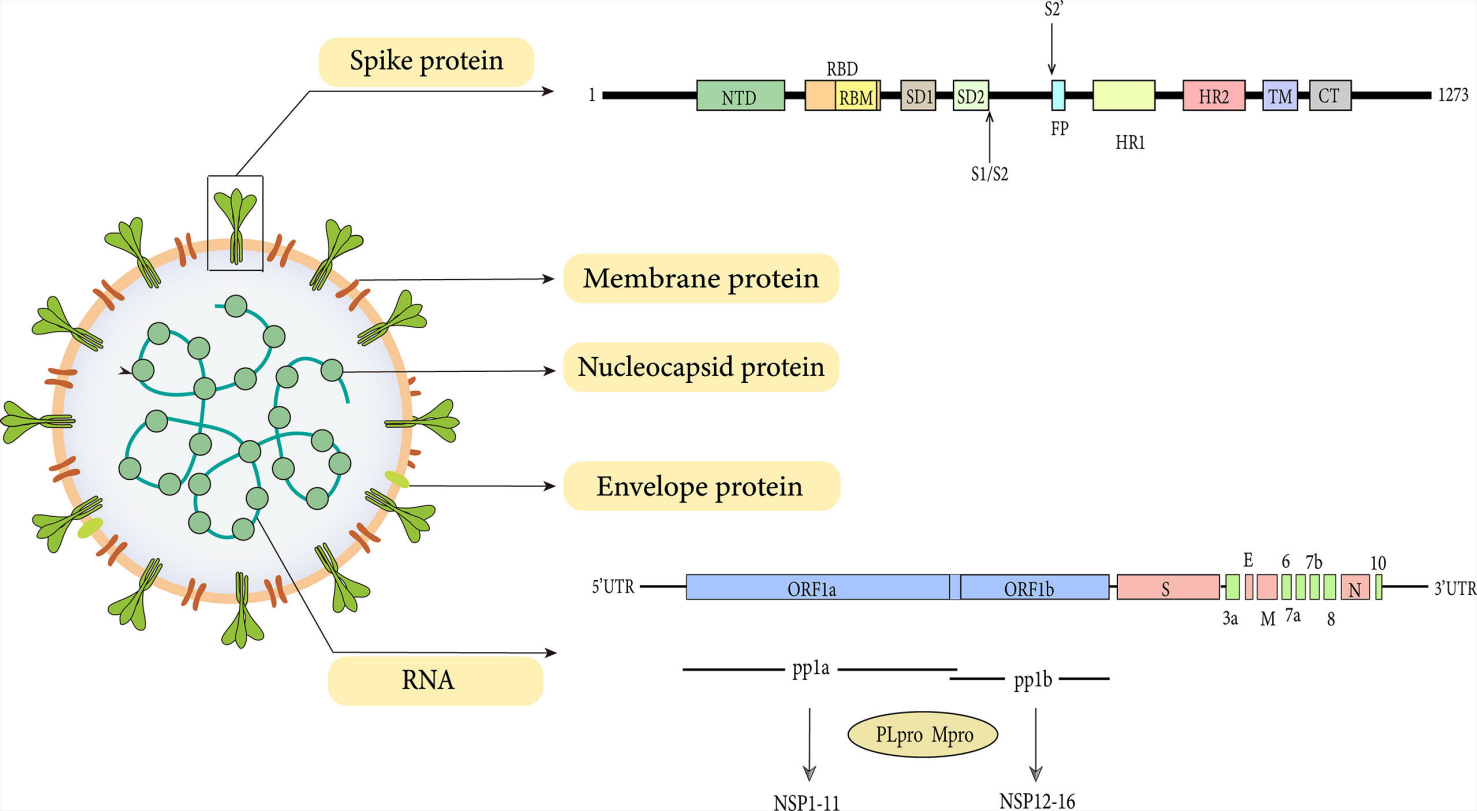
Genome and structure of SARS-CoV-2.

Transformation of the virus begins with the expression of ORF1a and ORF1b gene segments. ORF1a and ORF1b are encoded by the replicase gene and further translated into two large polyproteins (pp1a and pp1ab) (Plant and Dinman, 2008). The ORF1a gene encodes for pp1a protein, while ORF1b forms a fusion protein pp1a/b with pp1a through ribosomal frameshifting (Plant and Dinman, 2008). The processing products of pp1a are termed NSP1-NSP11, while NSP1-NSP10 and NSP12-NSP16 are processed products of pp1ab (**Figure 1**).

The translation frameshift site is a prominent feature of coronaviruses. It has been proposed that programmed ribosomal frameshifting, a crucial mechanism for regulation of ORF1b expression in coronaviruses, may depend on ribosome pausing (Lopinski et al., 2000). The slippery sequence (UUUAAAC) and a downstream hairpin-type pseudoknot help express proteins by causing a deformation of the reading frames (Bakhshandeh et al., 2021). Recently, it has been shown that the -PRRA- insertion sequence thought to be a translational pause site may be involved in the occurrence of stop codons (Postnikova et al., 2021). It has also been demonstrated that codon usage bias, which plays an important role in efficient RNA translation, determines programmed ribosomal frameshifting and ribosome pausing (Postnikova et al., 2021). Insertion of the furin site may be associated with the enhanced infectivity of SARS-CoV-2. This overlapping translation pausing is also strong evidence for the punctuated mode of evolution (Gould, 1994; Heasley et al., 2021). Furthermore, there are two variants (-HRRA- and -LRRA-), whose functional mechanism is not yet clear, that have recently been identified (Postnikova et al., 2021). Some believe that the -HRRA- probably impact infection and pathogenesis of the virus (Plante et al., 2021). Nevertheless, even furin’s overall impact on infectivity has recently been questioned, so this remains somewhat controversial (Papa et al., 2021; Johnson et al., 2021). Besides, since the frequency of the variant codon usage was not significantly altered, they may not drastically diminish the translation of the inserted sequence (Postnikova et al., 2021). Of course, it cannot be ruled out that other differences in the variants may affect the pathogenicity of the virus.

Structural proteins provide the structural elements of viral particles, while nonstructural proteins have a multi-faceted role in viral replication, transcription, morphogenesis, and evasion of host antiviral immune responses (Majumdar and Niyogi, 2021). Assembly of the replication-transcription complex (RTC) for cytoplasmic and membrane protection is pivotal to the formation of the new virus in host cells. Nsp3, one of the components of RTC, can be cleaved by PL protease to promote cytokine expression and suppress the host’s innate immune response (Bakhshandeh et al., 2021).

SARS-CoV-2 relies on RdRp, the central component of the replication/transcription mechanism, to replicate its genome, rather than host polymerase (Subissi et al., 2014; Gao et al., 2020). The NSP12 catalytic subunit, along with its auxiliary factors NSP7 and NSP8, constitutes the SARS-CoV-2 RdRp complex (Subissi et al., 2014). NSP12 can recognize templates, catalyze and extend nucleotide chains. The heterodimer composed of NSP7 and NSP8 acts as a cofactor not only to stabilize the complex but also to stimulate the enzymatic activity of NSP12 and enhance its binding to RNA. The second subunit of NSP8 is thought to play a role in extending the RNA template binding surface (Subissi et al., 2014; Zeng et al., 2021). Nucleotide incorporation errors caused by RdRp can be corrected by NSP14 (ExoN), a proofreading exonuclease, to improve the fidelity of RNA synthesis (Subissi et al., 2014).

Strains with RdRp mutations have been reported to have a 3-fold higher mutation rate than strains without RdRp mutations (Zeng et al., 2021). The mutation changes the hydrophobicity of the RdRp binding pocket, which may also be one of the reasons for the poor efficacy of remdesivir (Zeng et al., 2021).

### Structural Proteins

#### Spike Protein

As with all coronavirus, SARS-CoV-2 encodes four structural proteins which are the spike, envelope, matrix, and nucleocapsid (**Figure 1**). Despite the high structural similarity, the S protein of SARS-CoV-2 has a 10-20 folds stronger affinity for ACE2 than SARS-CoV, indicating that SARS-CoV-2 possesses more invasive ability (Wrapp et al., 2020). The structure of SARS-CoV-2 by cryo-EM has demonstrated that, like SARS-CoV, the spike protein of SARS-CoV-2 is also extensively glycosylated (Watanabe et al., 2020).

Spike protein (150 kDa) is a class I fusion protein. It is a large inactive precursor composed of 1273 amino acids and exists as a trimer on the surface of virion and has a characteristic crown-like appearance. The S protein is further processed into receptor‐binding subunit S1 and membrane‐fusion subunit S2, which have different functions respectively (Heald-Sargent and Gallagher, 2012). The S1 subunit consists of the signal sequence, N-terminal domain (NTD), and receptor binding domain (RBD) (**Figure 1**), where the receptor binding motif (RBM) binds to the ACE2 receptor to enter into the host cell (Shang et al., 2020). Furthermore, the binding can also lead to a conformational change in S2 subunit from a pre-fusion to a post-fusion state (Sainz et al., 2005). These changes favor the exposure and activation of the S2 domain “fusion peptide” which further promotes the fusion of viral and host cell membrane, thus initiating the process of endocytosis (Belouzard et al., 2009; Madu et al., 2009).

#### Envelop Protein

As stated earlier, the S-protein contributes to the entry of the virus inside the host cell while E-protein (8–12 kDa) is an integral transmembrane protein involved in virus assembly, budding, morphogenesis, and trafficking (Schoeman and Fielding, 2019). It is composed of three domains: a 7–12 amino acid short hydrophilic NTD, a 25 amino acid long hydrophobic transmembrane domain, and a lengthy hydrophilic C terminal region (Sarkar and Saha, 2020). Due to the ion channel activity of the hydrophobic domain, E protein also acts as a viroporin to facilitate viral release, in turn, pathogenicity (Nieto-Torres et al., 2014). By changing the ion homeostasis of cellular organelles, viroporins complete production, maturation, and release processes of the virus (Nieva et al., 2012). Noteworthy, according to the E protein sequence alignment, unlike SARS-CoV, the amino acid residue of SARS-CoV-2 at position 69 replaces the negatively charged glutamic acid with the positively charged arginine (Yoshimoto, 2020). We speculate that the substitution of positively charged basic amino acids for negatively charged acidic amino acids here may be associated with mutations that increase fitness and decrease virulence (Sun et al., 2020).The effect of this amino acid substitution on host range and immune evasion remains unknown.Besides, more research is required to further clarify whether this substitution affects the structure, function, and stability of E protein.

#### Membrane Protein

M protein (25–30 kDa) is the most abundant structural protein in the CoVs family, with three transmembrane domains that play critical roles in virion formation and complex stabilization during virion assembly by binding of a short glycosylated N-terminal portion of M protein to Nucleocapsid protein (Arndt et al., 2010). M protein gives the virus its spherical virion structure. In addition, research on a variety of CoVs revealed that the viral size is determined by the interaction of M protein with S, N proteins, and viral genomic RNA (Neuman et al., 2011). The M-protein is more abundant in coronaviruses than the E and S proteins, and it is conserved among β-coronaviruses (Ye and Hogue, 2007). Because of its interactions with all other structural proteins, the M protein is thought to be the fundamental organizer of viral assembly (Nieto-Torres et al., 2014). Interestingly, on the one hand, the interaction between M protein and S protein is needed for S protein retention in the ER-Golgi intermediate compartment and incorporation into new virions (Opstelten et al., 1995). M protein, on the other hand, is required for the intracellular production of viral particles that lack S protein. Coronavirus produces noninfectious virion with M protein but no S protein when Tunicamycin is present (Mousavizadeh and Ghasemi, 2021). Additionally, M-protein also appears to alter immune responses by blocking nuclear factor kappa B (NF-κB), resulting in viral multiplication (Fang et al., 2007). However, this theory is controversial. A recent study has demonstrated that the M protein of SARS-CoV-2, along with ORF3a, ORF7a, and N proteins, are considered NF-κB activators and do not directly inhibit or stimulate the IFN response (Su et al., 2021).

#### Nuleocaspid Protein

Nucleocapsid protein (N protein) is essential for the integration and packaging of viral genomic RNA into virions. It has multiple functions including RNA synthesis control, RNA packaging in helical nucleocapsids, and cooperating with the M protein to assemble virions (McBride et al., 2014). The gene of N protein is conserved and stable with little change over time, with 91 percent and 50 percent sequence identities to SARS-CoV and MERS-CoV, respectively (Hodge et al., 2021). The S, M, E proteins are responsible for the viral coat’s production, while the N protein is responsible for binding to the viral genome RNA and then condensing into a higher-order RNA-protein complex to initiate the assembly of virions, which is an essential step in the replication process of coronavirus.

In infected cells, N protein is produced in large amounts from sgRNA and is dynamically localized to the RTCs in the early stages of infection, where it promotes RNA template swapping and recruits host components to aid in the discontinuous transcription and translation of sgRNA (Verheije et al., 2010). The completion of the above functions depends on the characteristic modular structure evolved by the N protein. Similar to SARS-CoV, the N protein of SARS-CoV-2 is a 46 kDa protein that contains two conserved folding domains (the N-terminal RNA binding domain and the C-terminal dimerization domain), flanked by three intrinsically disordered regions (IDRs) (Peng et al., 2020). NTD mediates specific interactions with viral genome packaging signals, and CTD forms a compact dimer to facilitate vRNP assembly (Peng et al., 2020). A conserved core IDR with a serine/arginine-rich region separates two domains (SR). The degree of phosphorylation in SR can realize different functions by affecting the physical properties of N + RNA condensates (Lu et al., 2021).

RNA can trigger liquid-liquid phase separation (LLPS) of the N protein, which is a crucial step in viral assembly (Zhao et al., 2021). Acidic environments have been proven to facilitate the triggering process, so adjusting pH may also be a feasible antiviral treatment direction (Zhao et al., 2021). G3BP1 forms stress granules, which are an essential component of the host cell’s antiviral response, and LLPS mediates their formation (Sanders et al., 2020). The N protein interacts directly with co-localized G3BP1, possibly leading to sequestration of G3BP1, depletion of the cytoplasmic pool, and hindering the formation of stress granules to attenuate the stress response and evade the host innate immune response (Cubuk et al., 2021). Alternatively, the N protein might boost viral replication by hijacking this protein or stress granules (Hou et al., 2017). Additionally, the M protein can interact with N protein’s C-terminal region to independently mediate phase separation of N protein and promote the assembly of condensates without RNA (Neuman et al., 2011; Lu et al., 2021). RNA-mediated and M-mediated phase separation is achieved by forming condensates with distinct domains of the N protein (Lu et al., 2021). Individual vRNPs are assembled along the genomic RNA to form the packaging (Lu et al., 2021). Then the M protein interacts with these condensed RNPs and acts as an organizational hub for virion through a soluble CTD extending into the viral particle.

Taken together, proteins E, M, and N work together to assemble virions, whereas the S protein facilitates viral attachment, membrane fusion, and entrance (Mittal et al., 2020).

## Co-receptor

### ACE2

**Figure 2:**
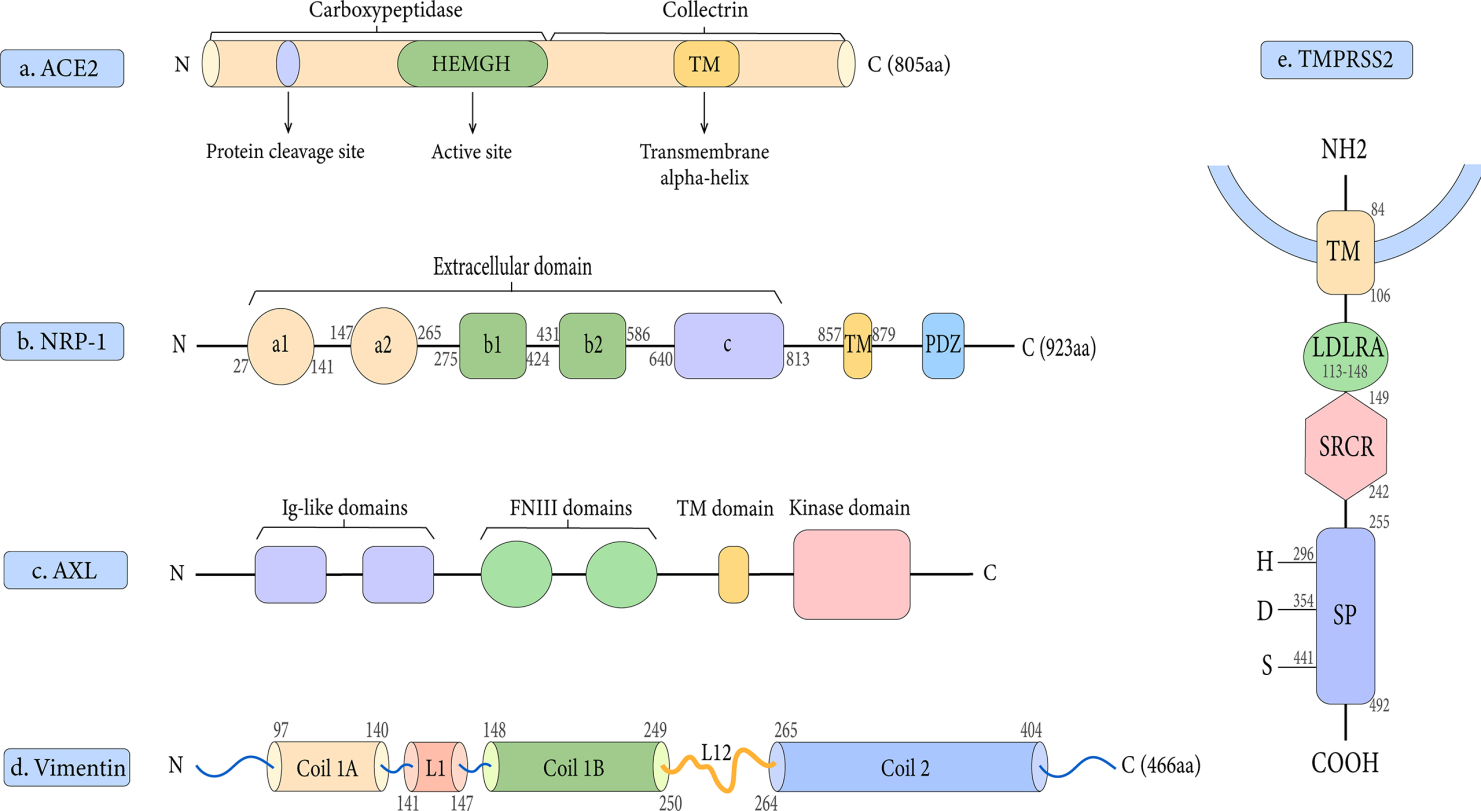
Schematic representation of the structures of the receptor and host proteases. **(A)** The structure of ACE2. **(B)** The structure of NRP-1. **(C)** The structure of AXL.**(D)** The structure of Vimentin. **(E)** The structure of TMPRSS2.

Early research has demonstrated that both SARS-CoV-2 and SARS-CoV employ ACE2 as a cell entrance receptor (Hoffmann et al., 2020). ACE2 is an integral membrane glycoprotein of type I that has a length of 805 amino acid residues (Jiang et al., 2014). It contains an N-terminal peptidase domain and a C-terminal collectrin-like domain (**Figure 2A**), ending with a 40 amino acid long single transmembrane intracellular segment (Yan et al., 2020). The C-terminal includes a transmembrane alpha-helix, while the N-terminal has one active site. There is also a signal peptide at the end of the N-terminal, in which the protein cleavage site is located next to it. As part of the renin-angiotensin system (RAS), ACE2 cleaves Ang I into Ang 1–9, which is then processed into Ang 1–7 (Yan et al., 2020). Angiotensin-(1–7), as a ligand, binds to the G-protein-coupled receptor MAS, which elicits responses that can counteract those of the ACE/angiotensin II/AT1 axis and exerts actions of vasodilation, vascular protection, anti-fibrosis, anti-proliferation, and anti-inflammation in multiple organs/systems (Patel et al., 2017; Santos et al., 2018). Moreover, ACE2 was found to have the functions of zinc metalloenzyme and carboxypeptidase on chromosome X (Beura et al., 2021). As a result, because men have only one X chromosome, they have a higher fatality rate from SARS-CoV-2 infection than women (Li et al., 2020).

After transcription, the N-terminal signal peptide is responsible for migration to the cell surface, while the C-terminal transmembrane domain is responsible for successful anchoring (Tipnis et al., 2000). The tip of one of the two lobes of the N-terminal peptidase domain binds to the spike RBD to initiate infection (Zhang et al., 2021).

### Neuropilin-1

Based on published findings, Neuropilin-1 is an important co-receptor for viral entry which allows SARS-CoV-2 and ACE2 to communicate more easily (Cantuti-Castelvetri et al., 2020). Neuropilin-1 (NRP-1) and Neuropilin-2 (NRP-2) are two members of the Neuropilin family that have a profound impact on lymphangiogenesis, angiogenesis, and axon guidance (Schellenburg et al., 2017). Both NRP-1 and NRP-2 are composed of five extracellular domains (a1/a2, b1/b2, and c domains), a single transmembrane (TM) stretch, and an intracellular PDZ domain-binding motif at C-terminal (Gu et al., 2002) (**Figure 2B**). Daly et al. showed that NRP-1 binds more strongly to the host cell surface than the S1 subunit, possibly destabilizing the S protein complex and activating the escape of S2 from the S1 subunit (Daly et al., 2020). Notably, there is proof that NRP-1 promotes SARS-CoV-2 penetration into the central nervous system (Daly et al., 2020). It has been suggested that this process may be through the olfactory epithelium of the nasal cavity, which perhaps explains the phenomenon of olfactory dysfunction seen in SARS-CoV-2 infected patients (Hopkins et al., 2021). In addition, our research group has studied the contribution of NRP-1 in the occurrence and development of liver fibrosis, which may provide new insights into the mechanism of SARS-CoV-2 in liver injury (Wang et al., 2019).

### AXL

AXL is a novel host receptor that not only enhances SARS-CoV-2 to enter human cells but also facilitates viral reproduction (Wang et al., 2021). It also known as Ark, UFO, or Tyro7, was originally a transforming gene isolated from human leukemia cells (O’ et al., 1991). As one of three receptor tyrosine kinases in the TAM family, it plays a key role in regulating cell growth, proliferation, apoptosis, and migration (Axelrod and Pienta, 2014). AXL consists of two immunoglobulin (Ig)-like repeats and two fibronectin type III (FN III)-like repeats, a transmembrane domain, and an intracellular kinase domain (Linger et al., 2008) (**Figure 2C**). One of the unique features of the interaction between AXL and the SARS-CoV-2 S protein is that AXL interacts with the S protein NTD rather than RBD (Wang et al., 2021). Also, AXL is considered an ACE2-independent receptor, given that it is not co-expressed with ACE2 in the human lung or trachea (Wang et al., 2021).

### Vimentin

Vimentin is a type III intermediate filament protein that is widely expressed on the outer surface of mesenchymal cells, including endothelial cells, fibroblasts, macrophages, melanocytes, and lymphocytes (Lowery et al., 2015). In human lung tissue, vimentin expression is high in lung endothelial cells, macrophages, T cells, and granulocytes, but low in type I and II alveolar cells and fibroblasts (Amraei et al., 2022). Vimentin is a 53 kDa polypeptide of 466 amino acids consisting of a central α-helical rod domain flanked by non-α-helical N and C-terminal domains (head and tail) (Danielsson et al., 2018). Its highly conserved α-helical rod domain has a Coil 1 motif near the N-terminus and a Coil 2 motif near the C-terminus (**Figure 2D**). Together, these molecules bind in parallel and align to form a coiled coil, which constitutes the basic structural building block of the entire filament protein family.

Endothelial cells have been recently identified as a direct target of SARS-CoV-2, and their damage can cause a transition from an anticoagulant to a procoagulant phenotype, resulting in microvascular thrombosis and severe coagulation disorders (Varga et al., 2020; Tang et al., 2020). In addition, it is expressed in type II alveolar cells and nasal goblet secretory cells, which also express the known receptors ACE2 and TMPRSS2 (Ziegler et al., 2020; Li et al., 2020). Interestingly, a novel mechanism has recently been proposed by which cells that do not express vimentin can acquire vimentin from the extracellular environment through neutrophil NETosis, a program of neutrophil death that accompanies the formation of neutrophil extracellular traps (NETs) (Khandpur et al., 2013; Thiam et al., 2020; Suprewicz et al., 2022). They also believe that vimentin may enable the SARS-CoV-2 virus to adhere to the cell surface, stimulate the cell membrane to wrap the virus, and thus initiate viral endocytosis (Suprewicz et al., 2022). Previous work has shown that vimentin can act as an attachment factor or coreceptor for SARS-CoV-1, Japanese encephalitis virus, cowpea mosaic virus, human papilloma virus, and dengue virus (Koudelka et al., 2009; Das et al., 2011; Yu et al., 2016; Yang et al., 2016; Schäfer et al., 2017). And it can directly interact with the S protein to enhance the entry of SARS-CoV during the binding process of the S protein to ACE2 (Yu et al., 2016). Amraei et al. showed that vimentin can bind to the RBD of SARS-CoV-2 as an attachment factor to facilitate viral entry and infection of endothelial cells (Amraei et al., 2022). Notably, different motifs on the RBD bind vimentin and ACE2 to improve ACE2-dependent viral entry, respectively (Amraei et al., 2022). Vimentin filaments can be found in the cytoplasm and extracellular compartments, and they co-localize with ACE2 in the cell-cell contact area, acting as a link for spike-ACE2 binding (Amraei et al., 2022).

In summary of earlier studies, vimentin plays important roles in viral infection and lung injury in the following ways: a. interacting with spike proteins to facilitate viral entry; b. influencing virus production during replication or assembly; c. participating in inflammatory and immune responses d. promoting endothelial mesenchymal transition and fibrosis (Li et al., 2020). But its specific role in SARS-CoV-2 virus infection needs more research to confirm. However, anti-vimentin antibodies will certainly be an effective therapeutic strategy for SARS-CoV-2 by blocking the infection of variants or reducing clinical symptoms. Meanwhile, vimentin-coated viruses may bind and aggregate with anti-vimentin antibodies, thereby inhibiting viral infection and promoting viral clearance.

### Other Co-Receptors

Besides all those mentioned above, there may be other receptors that mediate viral entry as research progresses. Several proteins that interact with SARS-CoV-2 S have been identified, including cellular heparan sulfate, sialic acids, CD147, and several C-type lectin receptors (DCL-SIGN, L-SIGN, MR, and MGL) (Clausen et al., 2020; Zhang et al., 2020; Gao et al., 2020; Wang et al., 2020; Thépaut et al., 2021; Sun, 2021). Because heparin significantly affects the open conformation of RBD making it more susceptible to ACE2 binding, cellular heparan sulfate plays an important role as a potential receptor in ACE2-dependent SARS-CoV-2 infection (Clausen et al., 2020). Sulfate and ACE2 binding sites on the RBD are next to each other, which provides the structural basis for this function (Clausen et al., 2020).

Furthermore, a receptor-overexpression and ligand-labeling system identified two additional potential candidate receptors, ASGR1 and KREMEN1, by screening more than 5000 human membrane proteins (Wang et al., 2021). They both interact with NTD and RBD, and KREMEN1 also interacts with the S2 domain. This diversity of receptor usage may explain why SARS-CoV-2 is more contagious than other coronaviruses.

## SARS-CoV-2 Interaction With Host Proteases

The coronavirus spike protein is always subjected to proteolysis happens with the assistance of numerous protease activators when binding to host cell receptors and initiating subsequent plasma membrane fusion or endocytosis (Li, 2016). In particular, viral fusion and entrance begin with exposure of the internal fusion peptide following proteolysis of the S protein (Zhang et al., 2021). The fusion and entry process requires three categories of proteases that function at different stages of infection: (a) phase of viral attachment: proprotein convertases (e.g., furin), (b) phase of S1 cleavage and detachment from the S2 domain: cell surface proteases [e.g., type II transmembrane serine protease (TMPRSS2)] and (c) phase of intraviral endocytosis: lysosomal proteases (e.g., cathepsin B/L) (Zhang et al., 2021). However, it should be pointed out that many extracellular proteases and many other host proteases may be involved in the cleavage of SARS-CoV-2 S protein based on the *in vivo* complexity and ubiquitous infection (Zhang et al., 2021).

### Furin

Furin is a calcium-dependent type I membrane-bound serine endoprotease enzyme that belongs to the seven-member family of subtilisin-like proprotein convertases (Thomas, 2002). Its main job is to complete the activation process by cutting off biologically inactive portions of the protein (Ganesan et al., 2020). SARS-CoV-2 inserts a polybasic residue (RRAR) at the junction of S1 and S2 cleavage sites, whereas SARS-CoV has only one basic amino acid (Amanat et al., 2021). Thus, the spike of SARS-CoV isn’t separated by proprotein convertases during virus particle formation and stays untouched on mature virions (Li, 2016). SARS-CoV-related coronaviruses (SARSr-CoV) lack this particular multibasic cleavage site, but it is present in human coronaviruses HKU1, OC43, and MERS-CoV (Hoffmann et al., 2020). Furin cuts S1/S2 site, granting the virus to infect host cells (Shiryaev et al., 2013). Unlike the restricted expression of other endoserine proteases, furin is extensively distributed and is expressed to different extents in distinct tissues and organs throughout the body (Ganesan et al., 2020). As a result, the almost ubiquitous expression of furin-like proteases may explain the high pathogenicity and severity, broad cell and tissue tropism, multiple organ damage as well as increasing its transmissibility of COVID-19 (Ganesan et al., 2020). Moreover, the spike cleavability is thought to determine the zoonotic potential under coronavirus infection (Hoffmann et al., 2020).

Recent studies have demonstrated that the addition of basic residues at the furin cleavage site in viral variants increases cell-cell fusion but not virus-cell fusion (Hoffmann et al., 2020). However, it should be mentioned that although the loss of furin significantly reduces infection by reducing the infectivity of virus particles rather than reducing virus production, it does not eliminate it (Papa et al., 2021). As a result, there is no doubt that furin is an extremely critical cofactor, but it is not required for infection, and replication will proceed even without it (Papa et al., 2021). This is also one of the reasons why existing antiviral treatments for SARS-CoV-2 based on furin-targeted medicines may not completely prevent viral infection (Papa et al., 2021). In addition, considering that furin is essential for normal development, short-term inhibitor treatment might be well-tolerated, but blocking this enzyme for a prolonged time might lead to unwanted toxic effects (Hoffmann et al., 2020).

### TMPRSS2

TMPRSS2 is a 492 amino acid residue long located on human chromosome 21q 22.3 (Antalis et al., 2011). It contains four different domains: a type II transmembrane domain, an LDL receptor class A (LDLRA) domain, a scavenger receptor cysteine-rich (SRCR) domain, and a serine protease domain (Thunders and Delahunt, 2020) (**Figure 2E**). To date, the physiological roles of TMPRSS2 are still not completely understood, but it is involved in a variety of biological processes (Thunders and Delahunt, 2020).

Another important function of TMPRSS2 is to cleave and trim the spike proteins to produce a better conformational state, and further activate the S protein to expose its fusion region to achieve the aim of ACE2 binding activity and virus-cell fusion (Hoffmann et al., 2020). It has been shown that TMPRSS2 expressing cells can isolate more SARS-CoV-2 virus particles than non-expressing cells (Matsuyama et al., 2020). It is characterized by a highly conserved catalytic serine protease domain stabilized by three intradomain disulphide bonds (Brooke and Prischi, 2020). The S1 domain contains the catalytic triad required for enzymatic activity, in analogy with the furin subtilisin-like domain (Brooke and Prischi, 2020). The catalytic triad binding site forms a negatively charged pocket, which is conducive to electrostatic interactions with the positively charged peptides of the Spike to catalyze the cleavage, leading to the viral entry (Brooke and Prischi, 2020; Abbasi et al., 2021). Unexpectedly, after the interaction of S protein with ACE2, TMPRSS2 cleaves at the arginine and lysine residues of ACE2, resulting inACE2 shedding and promoting viral particles uptake (Thunders and Delahunt, 2020). In addition to fusion mediated by virions, spike protein present at the plasma membrane can trigger the formation of receptor-dependent syncytia, and TMPRSS2 accelerates this process (Buchrieser et al., 2020). Furthermore, as an androgen-regulated gene, it may be one of the reasons for these gender disparities in the severity of disease course which persists across nations (Chakravarty et al., 2020). It is worth noting that loss of smell (anosmia), as one of the most prevalent and significant characteristics of COVID-19, may also be related to TMPRSS2 (Abbasi et al., 2021).

Indeed, it has been demonstrated that variants with loss of the TMPRSS2 cleavage site allow SARS-CoV-2 entry entirely *via* the endosome pathway and thus exhibit a more limited range of cell tropism (Lau et al., 2020; Sasaki et al., 2021). Under powerful selective pressure, these variants may exist at very low levels in some infected individuals, and it is necessary to screen more clinical samples of patients with mild or asymptomatic infections (Lau et al., 2020).

### Cathepsin B/L

Two ways can be used by coronaviruses to enter host cells: In the early stage of entry, the direct fusion of the virus on the plasma membrane is mediated by TMPRSS2; while in the late entry pathway, the coronavirus can be internalized *via* cathepsin-mediated endocytosis (Tang et al., 2020). Cathepsins are classified into three main categories: serine proteases cathepsins, aspartic proteases cathepsins, and lysosomal cysteine cathepsins (Tabrez et al., 2020). Endo- and exopeptidase cathepsin B and endopeptidase cathepsin L that play a critical role in endocytosis are lysosomal cysteine proteases (Hoffmann et al., 2020). The endoplasmic reticulum produces them, and the Golgi apparatus transports them to the lysosome and endosome (Zhang et al., 2021). Low pH is required for their optimal enzymatic activity due to their subcellular location (Zhang et al., 2021). Cathepsin B and Cathepsin L are known fusion activators that become active in early and late endosomes, respectively (Tang et al., 2020). Low endosome pH can activate cathepsin L to release the genome by triggering the fusion of virion membrane with endosome membrane (Tang et al., 2020). Evidence suggests that it is also cathepsin L, not cathepsin B, that plays a key role in the initiation of the S protein (Ou et al., 2020). We believe that elevating endosomal pH to inhibit the activity of cathepsin B/L or simultaneously targeting TMPRSS2 and cathepsin B/L are both feasible therapeutic directions.

## The Mechanism of SARS-CoV-2 Infection

**Figure 3:**
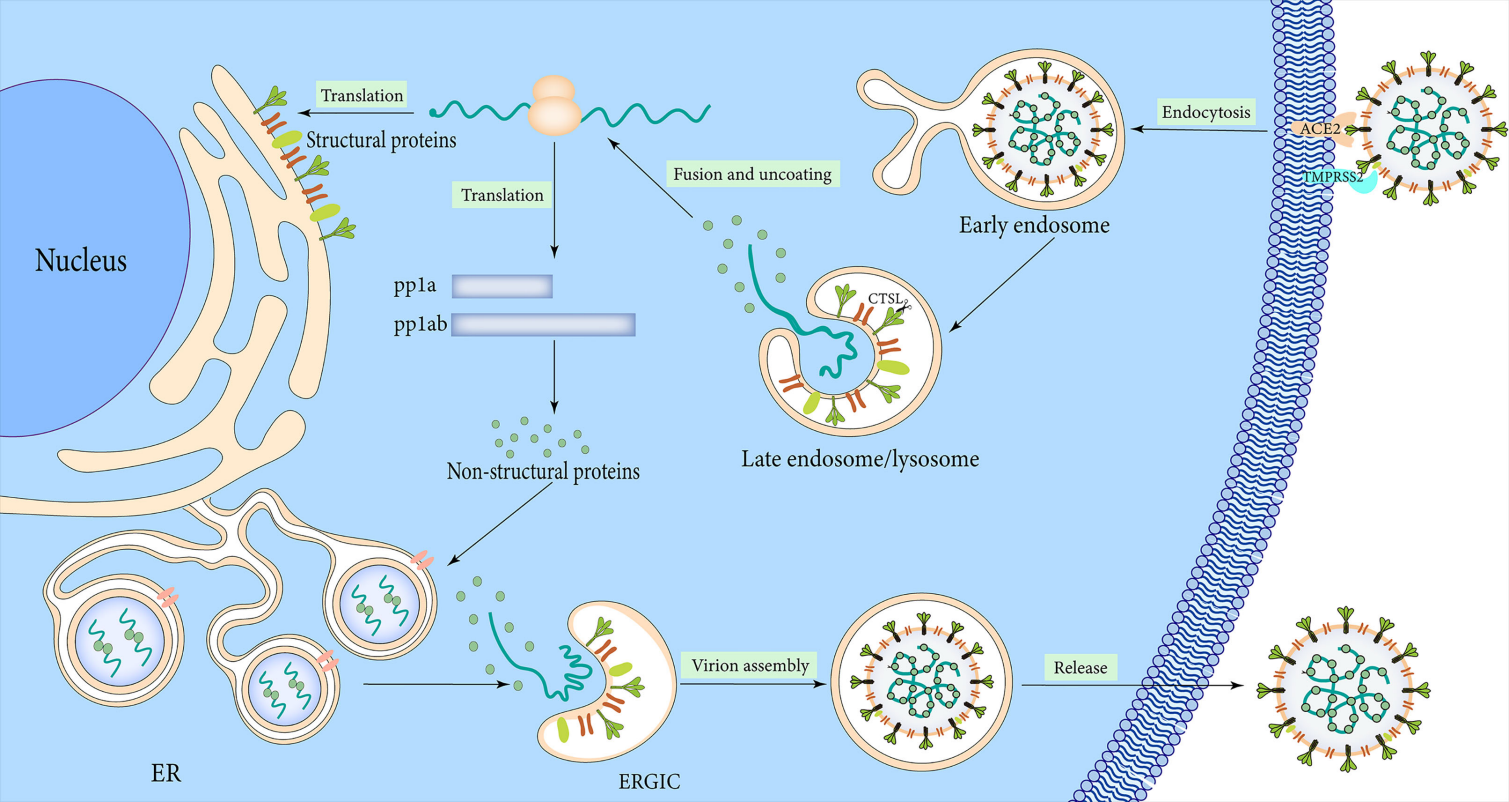
Infection mechanism of SARS-CoV-2.

The lifecycle of SARS-CoV-2 commences by binding the S1 RBD to the peptidase domain of ACE2 (**Figure 3**). Upon binding, furin cleaves S protein into the S1 and S2 subunits at the multibasic site, resulting in structural change in the S2 subunit (Walls et al., 2020). TMPRSS2 cleavage at the S2 site further exposes the fusion peptide (Walls et al., 2020). In the meantime, the S2 subunit’s heptad repeat 1 and heptad repeat 2 domains combine to produce a six-helix bundle fusion core, which brings the virus particles close to the host cell membrane (Xia et al., 2020). Cathepsins can work independently on cells lacking TMPRSS2 and form endocytosis and low pH endosomes, hence mediating virus-cell membrane fusion at the cell surface and endosomal compartments, respectively (Sasaki et al., 2021). Through either entry mechanism, the RNA genome is released into the cytosol, where it is translated into replicase protein and digested by chymotrypsin-like protease (3C*-*like protease or 3CL^pro^) and papain-like protease through a complicated multistep process to produce 16 non-structural proteins (Báez-Santos et al., 2015) (**Figure 3**). RdRp catalyzes viral genome replication and subgenomic transcription to assemble new viral particles.

The formation of double-membrane vesicles (DMVs) in the host cell induced by SARS-CoV-2 infection, is the first step in replication. NSP3 and NSP4 drive the rearrangement of the endoplasmic reticulum (ER) into DMV and promote genomic RNA (gRNA) and subgenomic RNAs (sgRNAs) replication (Tabata et al., 2021). The viral RNAs are stored in DMVs and transported to the cytosol for translation or through double-membrane-spanning pores for viral assembly (Zhang and Zhang, 2021). Structural proteins package gRNA to build progeny virus particles, while the shorter sgRNAs encode conserved structural and accessory proteins. The cytoplasm is the site of RNA and N protein synthesis, while S, M, and E proteins are synthesized in the endoplasmic reticulum and removed to the Golgi apparatus (**Figure 3**). From there, the viral RNA-N complex and S, M, and E proteins follow the secretory pathway to reach the endoplasmic reticulum-Golgi intermediate compartment (ERGIC) to assemble mature virions. After that, the virus particles are released through the budding process of the Golgi apparatus and exocytosis of the cell membrane to start a new round of infection. S protein monomer is also extensively modified *via* N-glycosylation and trimerizes in the ERGIC. The glycosylation of viral proteins has a wide range of roles, including mediating protein folding, affecting viral stability, infectivity, and immune evasion.

## The Emerging SARS-CoV-2 Variants

Variants that are currently spreading rapidly around the world can be divided into variants of concern, variants of interest, and variants under monitoring (WHO, 2022b). All variants share one specific mutation called D614G which was the dominant variant early in the global epidemic. The D614G mutation occurs when aspartic acid is replaced with glycine at position 614 of the S protein (Jackson et al., 2021). It was first detected in late January 2020, and began to emerge in March 2020, and continuously derived different clades (Jackson et al., 2021). The D614G mutation promotes allosteric of the RBD domain to the “up” conformation bound to the receptor ACE2 by eliminating the hydrogen-bonding interaction with T859 of the adjacent protomer from the spike trimer, thereby enhancing virion infectivity and further enhancing the replication of SARS-CoV-2 in the upper respiratory tract (Plante et al., 2021). In addition, due to the presence of D614G in the SD2 domain, it also enhances furin cleavage at the S1/S2 domain junction (Mohammad et al., 2021). However, the D614G proved to potentially reduce the binding affinity to ACE2 and possibly alter the predicted MHC binding, the biological significance of which warrants further investigation (Mohammad et al., 2021).

### Variants of Concern

Variants of concern (VOC) refer to VOI-defined variants with enhanced transmissibility, virulence, and poor response to current diagnostics, vaccines, and treatments (WHO, 2022b). The five variants of concern are alpha, beta, gamma, delta, and omicron.

#### Alpha (B.1.1.7 Lineage)

B.1.1.7 lineage was circulating in Britain as early as September 2020 and was identified in America at the end of December 2020 (Cascella et al., 2021). Seventeen mutations were identified in the Alpha variant genome. Among them, eight mutations (Δ69-70 deletion, Δ144 deletion, N501Y, A570D, P681H, T716I, S982A, D1118H) are in the spike protein. Amino acid changes in the B.1.1.7 protein improve both the accessibility of RBD and the affinity for ACE2, which might be one of the causes for the increased transmission (Cai et al., 2021). This N501Y single mutation increases the affinity between RBD and ACE2 by ~10-fold (Liu et al., 2021). Studies have shown that the N501Y mutation disrupts the stability of the SARS-CoV-2 S protein in addition to increase transmission rates (Mohammad et al., 2021). More seriously, however, individuals infected with the B.1.1.7 lineage variant have a significantly higher severity of disease and risk of death relative to those who infected with other variants (Challen et al., 2021).

#### Beta (B.1.351 Lineage or 20H)

The beta variant was reported in South Africa during mid-December 2020 and triggered a second wave of infections in Nelson Mandela Bay (Tegally et al., 2021). Beta variant has nine mutations (L18F, D80A, D215G, R246I, K417N, E484K, N501Y, D614G, and A701V) in the spike protein, of which three mutations (K417N, E484K, and N501Y) are found in RBD and enhance the affinity for the receptors (Cascella et al., 2021). The E484K mutation was shown to significantly alter the electrostatic complementarity of antibody binding to RBD (Mohammad et al., 2021). In comparison with the previous three largest lineages (B.1.1.54, B.1.1.56, and C.1) circulating in South Africa, B.1.351 shows remarkable hypermutation including nonsynonymous mutations that result in amino acid changes (Tegally et al., 2021). The N501Y substitution has also been identified in rapidly spreading lineages (B.1.1.7 and B.1.351), and was the only shared mutation of RBD among the three variants, indicating that exert significant functions in transmission and epidemic (Cascella et al., 2021; Tegally et al., 2021). Previous studies have shown that both N501Y substitution and E484K substitution may increase the affinity with human ACE2, and the combination of N501Y and E484K further enhances the affinity (Tegally et al., 2021).

Notably, the P71L amino acid substitution was discovered in beta variant’s E protein. And so far, no mutation of the E protein has been reported in all SARS-COV-2 variants except the Beta variant (Mohammad et al., 2021). The mutation is known to be associated with disease severity and mortality, but its specific mechanism needs further study. Although the full import of the mutations remains unclear, the genomic and epidemiological data demonstrate that this variant has a selective advantage as a result of greater transmissibility, immune escape, or both (Tegally et al., 2021).

#### Gamma (P.1 Lineage)

The gamma variant was reported in December 2020 in Brazil and was first identified in America in January 2021 (Cascella et al., 2021). The gamma variant includes ten mutations in the spike protein (L18F, T20N, P26S, D138Y, R190S, H655Y, T1027I V1176, K417T, E484K, and N501Y). Three mutations (L18F, K417N, E484K) are found in RBD, identical to the beta variant (Cascella et al., 2021).

#### Delta (B.1.617.2 Lineage)

The fourth variant of concern, delta variant is also known as the B.1.617.2 was initially identified in December 2020 in India and was responsible for the deadly second wave of COVID-19 infections in April 2021 in India (Cascella et al., 2021). In America, this variant was first reported in March 2021 and was reported to be more transmissible, surpassing preexisting variants of SARS-CoV-2 to emerge as the dominant SARS-CoV-2 variant in most countries (Cascella et al., 2021). The delta variant harbors ten mutations (T19R, (G142D*), 156del, 157del, R158G, L452R, T478K, D614G, P681R, D950N) in the spike protein (Cascella et al., 2021).

#### Omicron (B.1.1.529 Lineage)

The omicron variant, which has attracted the most global attention, was first reported in South Africa on November 23, 2021 and has been identified in over 100 countries and regions worldwide (Vaughan, 2021). The next day, WHO listed it as Variants under monitoring (VUM). On November 26, according to the evaluation results of the Virus Evolution Working Group, WHO named it omicron and listed it in VOC (Ferré et al., 2021). Preliminary data and analysis obtained locally show an exponential increase in the outbreak in South Africa, and in contrast to the prevalence of beta and delta variants, omicron reduces the risk of initial infection in the population but increases the risk of repeat infection (CDC, 2021).

Omicron has more than 50 mutations, 30 of which are on the S protein on the surface of the virus. Among them, there are more than 20 new mutations in the S1 domain, 8 mutations are located in NTD and 15 mutations are located in RBD, which may directly enhance the interaction between RBD and ACE2 and avoid binding to antibodies induced by previous infection or vaccination (Ferré et al., 2021). Mutations at the Flynn cleavage site may be linked to increased transmission (CoVariants, 2021). In addition, the insertion sequence (ins214EPE), which appeared for the first time in SARS-CoV-2, was shown to be expressed in the common cold coronavirus (HCoV-229E). This may explain the cold-like symptoms and short incubation period of around 3 days caused by omicron (CDC, 2021). Remarkably, however, other symptoms caused by omicron, such as loss of smell and taste, are not present in common influenza. Moreover, the long-term stay of the virus in the body can penetrate various organs. Even after recovery, it can still reignite and attack the CNS, causing many infected people to die of sequelae. Therefore, omicron will still have a serious impact in the long run.

Interestingly, traces of the omicron virus were found in wastewater in New York City on November 21, according to the latest traceability study published by the CDC (2022). This was the day before South African scientists announced the confirmation of the omicron variant and ten days before the first omicron variant infection was reported in the United States. Also in California and Texas, researchers found evidence of omicron in wastewater samples in late November (CDC, 2022). Besides, omicron variants have also been detected in wastewater treatment plants in France since mid-November (Ferré et al., 2021). Although the existing evidence cannot lead to the conclusive conclusion that omicron existed in these places at the time, it still has implications for the discovery and transmission of the virus.

### Variants of Interest

Variants of interest (VOI) are defined as variants with specific genetic markers that affect infectivity, disease severity, immune escape, diagnostic or therapeutic escape, and spread across multiple countries and regions causing serious damage. In the early stages, VOI consisted of eight variants, including Epsilon (B.1.427 and B.1.429); Zeta (P.2); Eta (B.1.525); Theta (P.3); Iota (B.1.526); Kappa (B.1.617.1); Lambda (C.37) and Mu (B.1.621) (Cascella et al., 2021). The first six variants have been reclassified as they have finally proven to no longer pose a significant risk to global public health. Therefore, the current VOI only includes Lambda and Mu variants (WHO, 2022b).

### Variants Under Monitoring

Variants under monitoring (VUM) are defined as a variant with genetic changes that may pose a risk in the future, requiring enhanced surveillance and repeated assessment (WHO, 2022b). The currently designated VUM mainly includes B.1.1.318, C.1.2, and B.1.640.

## Anti-viral Treatments of COVID-19

Under the severe situation of repeated outbreaks and the prevalence of new variants, there is still a lack of effective vaccines and antiviral treatments to control the spread of the epidemic, which is a huge challenge for us humans. A growing number of research based on the structure, function, infection mechanism and immunology of virus are pointing the way to the development of effective vaccines and specific drugs against variants in the future. Generally, antiviral therapy that inhibits the entry and replication of SARS-CoV-2 virus plays a significant role in the early stage of the disease; while in the later stage of the disease, when the immune/inflammatory response is enhanced, immunomodulatory and anti-inflammatory therapy may be more beneficial to patient’s recovery.

### Inhibit SARS-CoV-2 Entry

**Figure 4:**
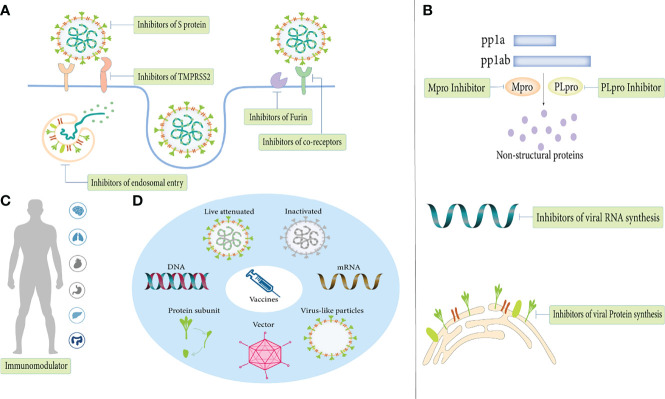
Anti-viral treatments of COVID-19. **(A)** Inhibit SARS-CoV-2 entry. **(B)** Inhibit SARS-CoV-2 replication. **(C)** Immunomodulator. **(D)** Vaccines.

The first step in the infection process is the attachment and entry of SARS-COV-2 into host cells, and blocking this process has critical implications for disease prevention and early treatment (**Figure 4A**). Blocking the S protein that binds to the receptor is essential for suppressing infection. At present, S protein inhibitors mainly include monoclonal antibodies, convalescent plasma, nanobodies, miniproteins, human soluble ACE2 and ACE2 receptor trap molecules (Rojas et al., 2020; Esparza et al., 2020; Cao et al., 2020; Monteil et al., 2020; Linsky et al., 2020; Corti et al., 2021). For receptors, lactoferrin blocks the attachment of virus to the host cell by binding heparan sulfate, and can synergize with remdesivir to exert an anti-viral effect (Hu et al., 2021). As research progresses, more co-receptors are identified and are expected to become drug targets for COVID-19 therapy. Inhibitors of co-receptors and their generic particles, while in principle suppressing SARS-CoV-2 infection, still need to be validated in more clinical trials.

SARS-CoV-2 enters host cells *via* receptor-mediated membrane fusion or the endosomal pathway. Host proteases (eg, furin, TMMPRS2) can promote S protein attachment and virus-cell membrane fusion, so their inhibitors are essential to block fusion entry of SARS-COV-2. Furin inhibitors (decanoyl-RVKR-chloromethylketone) and TMPRSS2 inhibitors (camostat mesylate, neformastat, antiandrogens, bromhexine) have been shown to have varying degrees of effect in reducing SARS-COV-2 infection (Zhou et al., 2015; Hoffmann et al., 2020; Mollica et al., 2020; Ansarin et al., 2020; Cheng et al., 2020). In addition, Catepsin L inhibitors (SSAA09E1, teicoplanin, K1777), hydroxychloroquine, umifenovir, nitazoxanide and niclosamide all exert antiviral effects through the endosomal entry pathway (Axfors et al., 2021; Şimşek-Yavuz and Komsuoğlu Çelikyurt, 2021; Huang et al., 2021; Lokhande and Devarajan, 2021; Backer et al., 2021).

### Inhibit SARS-CoV-2 Replication

After viral entry, inhibition of viral replication by inhibiting viral RNA and protein synthesis is another mechanism in antiviral therapy (**Figure 4B**). M^pro^ and PL^pro^ are attractive targets for drug development as proteases essential for viral replication. As M^pro^ is highly conserved and has no human homologues, its inhibitors (lopinavir/ritonavir, PF-07321332, PF-07304814, GC376) are currently in various stages of preclinical and clinical development (Şimşek-Yavuz and Komsuoğlu Çelikyurt, 2021). Inhibitors that inhibit RNA synthesis mainly include RdRp inhibitors (remdesivir, favipiravir, molnupiravir, AT-527), RNA synthesis-related host protein inhibitors (merimepodib and PTC299) and a gene-editing enzyme, Cas13a, that shreds RNA into pieces (Luban et al., 2020; Şimşek-Yavuz and Komsuoğlu Çelikyurt, 2021; Adegbola et al., 2021; Blanchard et al., 2021). In contrast to targeting viral proteins, targeting host proteins has multiple advantages. Precisely, because of such a high rate of mutation of the virus, anti-viral drugs will quickly lose their effectiveness, but due to the relatively host low mutation rate, targeting the host protein is a more feasible therapeutic strategy (Azouz et al., 2020). Host protein inhibitors that support viral protein synthesis including the eEF1A inhibitor (plitidepsin) and the ER chaperon protein inhibitor (fluvoxamine) exhibit potent antiviral activity (Şimşek-Yavuz and Komsuoğlu Çelikyurt, 2021). Of note, targeting human proteins is the potential risk of changing the physiological pathway. In conclusion, more study is required to definitively comprehend the specific effects of host proteins on the human body to guide the feasible direction of future antiviral therapy.

### Immunomodulator

Immune responses play a key role in disease process, and the immune escape mechanisms of SARS-COV-2 and variants are not fully understood. Immunomodulators such as ivermectin and interferon have demonstrated antiviral activity in a variety of viruses, but their therapeutic effects on COVID-19 are still controversial (**Figure 4C**) (Caly et al., 2020; Alavi Darazam et al., 2021). Additionally, traditional Chinese medicine also has immunomodulatory effects, and can fight viruses independently or in synergy with Western medicine (An et al., 2021). Due to the lack of relevant studies, whether there are other antiviral mechanisms besides immune regulation remains to be further elucidated. Furthermore, in December 2021, China developed the first COVID-19 specific antibody drug, the combination therapy of BRII-196/BRII-198, and approved for marketing. Clinical data show that the antibody can remain in the human body for 9 to 12 months. It is active against the main popular variants and plays a certain role in preventing infection. On March 14, 2022, the drug was included in the ninth edition of China’s COVID-19 diagnosis and treatment plan.

### Vaccines

In the near future, newly developed vaccines are also expected to protect against existing and emerging variants of SARS-CoV-2 (**Figure 4D**). According to statistics, as of March 2022, there are 340 candidate vaccines in the world, with 122 in the clinical trial stage and 30 vaccines in routine use (LSHTM, 2021). The types of SARS-CoV-2 vaccines mainly include inactivated vaccines, live attenuated vaccines, vector vaccines (replicating and non-replicating vectors), protein subunit vaccines, virus-like particle vaccines, DNA vaccines, RNA vaccines and other unknown types. Among the three mainstream vaccines currently used in worldwide, RNA vaccines are the most effective, followed by viral vector vaccines and inactivated virus vaccines (Ling et al., 2021). However, inactivated vaccines have the lowest incidence of adverse events, and the safety of mRNA vaccines and viral vector vaccines remains controversial.

## Conclusion and Perspectives

Viral genomes have evolved due to mutations that allow them to adapt well to their hosts and reproduce continuously. They make the virus more infectious, transmissible and help evade the host’s immune response by modifying the epitope of the gene. As the COVID-19 pandemic develops, a deeper study of those evolving SARS-CoV-2 variants is critical to understanding mutational adaptability and identifying control measures for the COVID-19 pandemic. A global survey of SARS-CoV-2 genes revealed that mutations in structural, nonstructural, accessory proteins, and untranslated regions were the most common (Majumdar and Niyogi, 2021). Furthermore, mutations in ORF1a, ORF1b, N, and S proteins were present in almost all countries, with the least number of variants in M and E, indicating that they are conserved proteins (Majumdar and Niyogi, 2021). Single nucleotide substitutions are the most common of the numerous forms of mutations. Additionally, insertions, deletions, and frameshift mutations have also been reported, albeit at lower frequencies.

Mutations can alter the antigenic properties of glycoproteins through a variety of different mechanisms, including increasing receptor binding affinity, deleting or inserting residues, altering epitope amino acid substitutions, glycosylation motifs, and protein conformation. Emerging SARS-CoV-2 variants share common features: increased virus transmissibility, infectivity, virulence, and antibody resistance from convalescent sera or vaccines, while also evolving the ability to immune escape. However, the emergence of attenuating mutations suggests an evolutionary trend toward reduced pathogenicity to achieve long-term coexistence with the host. Many scholars believe that the omicron variant is expected to end the COVID-19 pandemic, or at least reduce the impact on life in the future. A group of studies in South Africa argues that the omicron variant is highly contagious and can quickly replace the more pathogenic delta as the dominant variant in various countries (Khan et al., 2021). And, because it causes mild symptoms, the body produces enough neutralizing antibodies that when we encounter the more lethal variants, there will be no secondary infections. Reducing the pathogenicity of SARS-CoV-2 as well as improving immunity in the population may lead to a reduction in critical cases, resulting in a significantly weakened pandemic. Nonetheless, there is still very limited information on the current status of omicron, such as genomics, transmissibility, vaccine efficacy, treatment, and management. Some researchers hold different attitudes towards its future development because from the current evidence, the infectivity, pathogenicity, and immune escape of omicron have been comprehensively strengthened than previous variants, which may bring more serious consequences.

Another worrying fact is that on January 7, 2022, virologists from the University of Cyprus discovered a new variant with both delta and omicron mutations, and named it deltacron. At present, the variant has been found in countries such as France, the Netherlands and Denmark, and the WHO has also confirmed that deltacron is real and not the result of contamination of laboratory samples (Philippe et al., 2022; Kreier, 2022). Due to the small number of cases and unknown characteristics of the new variant, it is not classified as a VOC by the WHO for the time being. However, whether deltacron can be both highly pathogenic as the delta variant and highly transmissible as the omicron variant is a matter of global concern. Judging from the current situation, SARS-CoV-2 is still spreading rapidly around the world, the pandemic is far from over, and the future direction of the epidemic is still confusing. Therefore, better knowledge of how mutations affect people in the SARS-CoV-2 genome and its adaptation to the host will help to elucidate the drivers of transmission and evolutionary success. It is worth noting that an article published by Nature on January 12, 2022 elaborates a new perspective on evolution. Existing theories hold that mutations are completely random, and that natural selection determines which mutations survive. However, they found that mutations in plants are somewhat non-random and that essential genes with important biological functions have a much lower mutation frequency (Monroe et al., 2022). This adaptive mutational bias is a product of evolution and may vary between organisms, a finding that adds a surprising twist to Darwin’s theory of evolution by natural selection. Although this theory has not been tested in other species, it may offer another explanation for many of the observations in viral mutations. Since this mutational bias in plants is to protect key genes to ensure survival, we wondered whether high-frequency mutations in key structures and key sites in viruses are also adaptive mutational biases that are forced by survival. As research continues to deepen and the knowledge base expands, we believe that mysteries about viruses and their variants will be revealed one by one.

## Author Contributions

Conception and design: HB and FL. Collection and assembly of data: JL, HJ, MT, NW, JQ, XY. Data analysis and interpretation: JL, NW, XY. Manuscript writing: JL, HB and FL, JQ. Administrative support: WR and JQ. All authors contributed to the article and approved the submitted version.

## Funding

This work was supported in part by grants from the Clinical Medical Science and Technology Innovation Program (202019094), the Natural Science Foundation of Shandong Province (ZR2021MH139) and WBE Liver Fibrosis Foundation (CFHPC2021011).

## Conflict of Interest

The authors declare that the research was conducted in the absence of any commercial or financial relationships that could be construed as a potential conflict of interest.

## Publisher’s Note

All claims expressed in this article are solely those of the authors and do not necessarily represent those of their affiliated organizations, or those of the publisher, the editors and the reviewers. Any product that may be evaluated in this article, or claim that may be made by its manufacturer, is not guaranteed or endorsed by the publisher.
